# Jump Squats Performed with Both Light and Heavy Loads Have Similar Effects on the Physical Performance of Elite Rugby Players during the Initial Phase of the Competitive Period

**DOI:** 10.5114/jhk/186340

**Published:** 2024-04-15

**Authors:** Irineu Loturco, Lucas A. Pereira, Túlio B. M. A. Moura, Valter P. Mercer, Marina T. Betelli, Maurício S. Ramos, Santiago Zabaloy, Fernando Pareja-Blanco

**Affiliations:** 1 NAR—Nucleus of High Performance in Sport, São Paulo, Brazil.; 2 Department of Human Movement Sciences, Federal University of São Paulo, São Paulo, Brazil.; 3 Department of Sport, Health, and Exercise Science, University of South Wales, Pontypridd, Wales, United Kingdom.; 4 CBRu—Brazilian Rugby Confederation, São Paulo, Brazil.; 5 Faculty of Physical Activity and Sports, University of Flores, Buenos Aires, Argentina.; 6 Physical Performance & Sports Research Center, Department of Sports and Computer Sciences, Universidad Pablo de Olavide, Seville, Spain.; 7 Faculty of Sport Sciences, Department of Sports and Computer Sciences, Universidad Pablo de Olavide, Seville, Spain.

**Keywords:** sports, sprint velocity, muscle power, athletic performance, resistance training

## Abstract

We examined the effectiveness of two different jump-squat (JS) loading ranges on the physical performance of rugby players. Twenty-eight elite male rugby players were divided into two JS training groups: a light-load JS group (“LJS”; JS at 40% of the one-repetition maximum [1RM] in the half-squat (HS) exercise) and a heavy-load JS group (“HJS”; JS at 80% HS-1RM). Players completed the distinct training programs over four weeks, three times per week, during the initial phase of the competitive period. Pre- and post-training tests were conducted in the following sequence: vertical jumps, a 30-m speed test, peak power in the JS and the HS, and maximum isometric force in the HS. Additionally, the rating of perceived exertion (RPE) was assessed at the end of all training sessions throughout the intervention. A two-way ANOVA with repeated measures, followed by the Tukey’s post-hoc test, was employed to analyze differences between groups. The level of significance was set at p < 0.05. Effect sizes were used to assess the magnitude of differences between pre- and post-training data. Except for the RPE values (which were lower in the LJS group), no significant changes were detected for any other variable. In summary, using either a light- (40% HS-1RM) or a heavy-load (80% HS-1RM) JS during the initial phase of the competitive period is equally effective in maintaining physical performance levels attained during the preceding training period (pre-season), with the significant advantage of the light-load protocol resulting in lower levels of the RPE. This finding may have important implications for resistance training programming, especially in disciplines where acute and chronic fatigue is always a problematic issue.

## Introduction

Resistance training programming and prescription are among the most commonly researched topics in sport science (Kraemer and Fleck, 1988; Loturco et al., 2024; Williams et al., 2017). Indeed, the appropriate development of neuromuscular qualities is highly relevant in the vast majority of sport disciplines, whether they are directly related to strength-power performance (e.g., sprint events, team-sports, etc.) (Cronin and Sleivert, 2005; Krzysztofik et al., 2023; Loturco et al., 2024; Suchomel et al., 2016) or even those more associated with endurance capabilities (e.g., triathlon and long-distance running) (Denadai et al., 2017; Suchomel et al., 2016). In general, coaches and researchers are always seeking more efficient ways of maximizing the performance gains provided by resistance training programs, while also being interested in avoiding or at least reducing fatigue accumulated throughout the entire training season (Dubois et al., 2020; Twist and Highton, 2013). In this regard, it is important to emphasize that resistance training sessions, especially when prescribed at “high doses”, combined with sport-specific training sessions and competitions, may result in higher levels of acute and chronic fatigue (Draganidis et al., 2013; Grainger et al., 2023; Sanchez-Medina and González-Badillo, 2011). This, in turn, increases the risk of non-functional overreaching, muscle pain, as well as injuries, and may be detrimental to athletic performance (Moreno-Villanueva et al., 2022; Ribeiro et al., 2022; Thorpe et al., 2017).

Among all training variables (e.g., volume, the exercise type, frequency, etc.), loading intensity (e.g., percentage of one-repetition maximum [%1RM]) is undoubtedly one of the most examined and influential factors in resistance training (Haff, 2010). Different studies employing similar training programs, methods or exercises were designed to analyze the effects of various loading conditions (e.g., light vs. heavy loads; “optimum” (typically, moderate loads; 45–60% 1RM) vs. lighter or heavier loads, etc.) on the physical performance of subjects with distinct training backgrounds. For example, Mcbride et al. (2002) compared chronic adaptations resulting from two different eight-week jump squat (JS) training regimes performed at 30% or 80% of participants’ 1RM in the half-squat (HS) exercise on the neuromuscular performance of twenty-six men with varying levels of resistance training experience. In terms of strength and power qualities, both the JS30 and JS80 groups demonstrated significant increases in peak power at 55% and 80% 1RM, as well as in the 1RM value. However, only the JS30 group exhibited significant improvements in peak power and peak velocity across the entire loading range (i.e., from 30% to 80% 1RM) and showed a trend towards increased sprint speed at 20 m. Notably, and somewhat surprisingly, the JS80 group showed a significant decrease in 5-m sprint speed. This could pose a significant challenge for team-sport disciplines, where athletes must predominantly and effectively execute short sprints (≤ 10–20 m) during technical-tactical training sessions and matches (Faude et al., 2012; Gabbett, 2012).

The discussion regarding training at higher speed-lower strength versus training at higher strength-lower speed is not new and frequently involves the development of novel and alternative methods and techniques for strength-power training. The use of assisted exercises (e.g., lifts performed with the aid of elastic bands) is, for example, one of the strategies employed by coaches to artificially increase movement velocity (Loturco et al., 2015, 2024). A study with elite young soccer players (Loturco et al., 2015) revealed that, even when they executed ballistic movements with light loads (e.g., JS at 40% body mass (BM)), the increases in sprint speed were greater in athletes who trained under increased velocity conditions (i.e., using an elastic band system able to increase bar velocity by 20%). In contrast, although both groups presented significant improvements in maximum dynamic strength, the JS 40% BM group exhibited superior gains in 1RM strength. An important aspect in the former study is that, regardless of the differences in movement velocities (i.e., increased and decreased velocity groups trained with a mean difference of 40% in bar velocity), both groups were required to move the barbell as fast as possible during the JS executions, thereby applying the maximum force possible against the barbell (Loturco et al., 2015, 2022b; Valenzuela-Barrero et al., 2023).

Other studies have compared the effectiveness of different loading intensities under similar training schemes and obtained comparable results. Freitas et al. (2019) examined the effects of two distinct complex training protocols in terms of the load prescription: an optimal load (OL) training versus a modified complex training protocol (i.e., HSs, bench press exercises, and hip thrusts executed at the OL (~30–60% 1RM) versus the OL + 80% 1RM, respectively) and obtained very similar training responses. Research conducted with athletic and non-athletic populations, who trained within diverse loading ranges and followed similar training protocols (i.e., similar exercises and total training volume) also demonstrated that JSs with heavy loads (i.e., 80% 1RM) or the combination of back squats and JSs executed across a wide range of loads (i.e., from 30% to 80% 1RM) were not more effective than equivalent training programs performed at light-to-moderate loads (i.e., ~20–65% 1RM) in increasing absolute strength values and speed qualities (Harris et al., 2008; Loturco et al., 2013). The effects of light versus heavy loads were also analyzed in tapering studies conducted with elite athletes. Zaras et al. (2014) compared the effects of two 2-week taper phases, during which track and field athletes (i.e., throwers) completed very similar tapering programs at 30% 1RM (light-load taper) or 80% 1RM (heavy-load taper) and observed similar improvements in throwing performance, with mean increases of approximately 5% in shot put, javelin, hammer, and discus throwing distances. Notwithstanding the greater increases in the leg press 1RM and JS power detected in the heavy-load taper group, the light-load tapering was significantly easier to perform, resulting in lower levels of perceived fatigue, as assessed by the rate of perceived exertion (RPE), immediately after the end of the 2^nd^ tapering phase (Zaras et al., 2014) (which may represent an important advantage during certain points of the competitive period) (Gonçalves et al., 2020). Considering all aspects and scenarios listed above, it would be interesting to examine the effectiveness of different loading conditions (i.e., light vs. heavy loads) under similar training regimes, specifically incorporating ballistic exercises (e.g., a JS) in a real-world context routine throughout the competitive period in elite athletes with elevated levels of strength and power and an extensive resistance training background. Therefore, the aim of this study was to compare the effects of the JS executed at 40% or 80% HS-1RM on strength, speed, and power performance of national team rugby players during the initial phase of the competitive period.

## Methods

### Participants

Twenty-eight male rugby union players from the Brazilian national team (age: 25.4 ± 2.7 years; BM: 94.5 ± 16.4 kg; body height: 1.82 ± 0.15 m; 1RM relative to BM: ≥ ~2) participated in this study. Athletes were randomly allocated to one of the two training groups: a light-load JS group (i.e., LJS; n = 14); and a heavy-load JS group (i.e., HJS; n = 14). Players’ names were entered in order from the lowest to the highest 30-m sprint time by an independent researcher in a customized spreadsheet and grouped in pairs according to their baseline results. Subsequently, the group allocation of each pair was determined by tossing a coin. Three athletes did not complete all training sessions and were excluded from the analysis. Thus, the final dataset included 25 athletes (LJS; n = 14 and HJS; n = 11). The research was approved by the Ethics Committee of the Federal University of São Paulo (protocol code: 4.355.629; approval date: 22 October 2020), and all participants signed an informed consent form prior to the study commencement.

### Design

This parallel, two-group study was designed to test the effects of two training programs executed under different loading conditions (i.e., light vs. heavy) on the strength, speed, and power performance of elite rugby union players during a 4-week training phase, completed within the initial phase of the competitive period, comprising one friendly and two official rugby matches. Table 1 shows the typical weekly training and competitive schedule of players across the intervention. The resistance training program followed by athletes during this period is presented in Table 2. Training content (i.e., frequency, volume, exercise types, and intensity) was defined and determined in conjunction with the coaching staff of the Brazilian national team, respecting their principles, habits, and routines. They performed 12 resistance training sessions over the four weeks. The LJS group performed a JS with a load corresponding to 40% of the HS-1RM, while the HJS group performed a JS at 80% HS-1RM. Except for the differences between JS loading intensities (i.e., 40% vs. 80% HS-1RM), players from both groups followed the same training and match routines throughout the study. All athletes were previously familiarized with training and testing procedures. Prior to all testing sessions, a general and specific warm-up was completed, involving light-to-moderate running for 10 min followed by dynamic stretching and submaximal attempts of each tested exercise. The pre- and post-training tests were conducted in the following order: squats and countermovement jumps (SJs and CMJs), 30-m sprints, progressive loading tests to determine peak power (PP) in the JS and HS exercises, and maximum isometric force (MIF) in the HS exercise. Post-training measurements were conducted at the end of week 4, and during the subsequent weekend, before the post-tests, athletes had a 48-h rest interval without engaging in any training activities. Finally, the rate of perceived exertion (RPE) was assessed after every resistance training session during the entire intervention.

**Table 1:** A typical training program for elite rugby players over the 4-week training period.

Monday	Tuesday	Wednesday	Thursday	Friday	Saturday
Resistance Training 60’ RSCond Training 30’ TEC/TAC 90’	Resistance Training 60” TEC/TAC 120’	RSCond Training 30’ TEC/TAC 120’	Resistance Training 60’ RSCond Training 30’ TEC/TAC 90’	TEC/TAC 60’	1 FM and 2 OM 80’

*Note:* TEC = technical training; TAC = tactical training; numbers after the training session represent the volume (training duration) in minutes. RSCond refers to rugby-specific conditioning training, which incorporates various formats of high-intensity interval training and drills. TEC/TAC training comprises different formats of game-based drills and specific technical-tactical actions (e.g., pass drills, line-out formation, scrum simulation, etc.). Throughout the study period, one friendly match (FM) and two official matches (OM) were played on Saturday.

**Table 2:** The resistance training program of the elite rugby players during the first phase of the competitive period (4 weeks).

Exercise		Sets	Repetitions	Load
**Day 1**	Jump-squat	4–6*	6	40% or 80%#^1^RM
	Push press	3	8	60–70% 1RM
	Hang high pull	3	8	60–70% 1RM
	Parallel dips	3	8	BM
	Reverse fly	3	6–8	70–80% 1RM
	Lateral raises	2	6–8	70–80% 1RM
**Day 2**	Jump-squat	4–6	6	40% or 80% 1RM
	Stiff-leg deadlift	3	6–8	70–80% 1RM
	Bench press	3	4–8	80–90% 1RM
	Bench press 45o	3	4–8	80–90% 1RM
	Fly	2	8 6	60–70% 1RM
	Nordic	2		BM
**Day 3**	Jump-squat	4–6	6	40% or 80% 1RM
	Push up	3	8	BM
	Unilateral row	3	6–8	70–80% 1RM
	Prone row	3	4–8	80–90% 1RM
	Lumbar extension	3	20	BM

* In sessions 1–4 and 9–12, players executed 4 sets, while in sessions 5–8 they performed 6 sets. ^#^ Players were randomly divided into two groups: the light-load jump-squat group (LJS), which executed jump squats at 40% of the half-squat one-repetition maximum (HS-1RM); and the heavy-load jump-squat group (HJS), which executed jump squats at 80% HS-1RM. BM: body mass.

### Procedures

#### Session Rating of Perceived Exertion

The RPE was assessed 30 min after the completion of each of the 12 resistance training sessions throughout the study. Athletes were required to report the intensity of training sessions by means of a 10-point RPE scale (Foster et al., 2001).

#### Vertical Jump Tests

Vertical jump height was assessed using the SJ and the CMJ. In the SJ, a static position with a ~90° knee flexion angle was maintained for 2 s before a jump attempt without any preparatory movement. In the CMJ, players were instructed to perform a downward movement followed by complete extension of the lower limbs, and the amplitude of the countermovement was freely determined to avoid changes in the jumping coordination pattern (Pereira et al., 2022a). All jumps were executed with hands on the hips. Five attempts of each jump were performed interspersed by 15-s intervals. The jumps were performed on a contact platform (Elite Jump System®; S2 Sports, São Paulo, Brazil), and the best result of each jump was used for further data analysis.

#### Sprinting Speed

Sprint testing was conducted on an indoor running track. Two pairs of photocells (Elite Speed System®; S2 Sports, São Paulo, Brazil) were positioned at the starting line and at a distance of 30 m. Players sprinted twice, starting from a standing position 0.5 m behind the starting line. Sprint speed was calculated as the distance travelled over a measured time interval. A 5-min rest interval was allowed between trials, and the fastest time was considered for analysis.

#### Maximum Isometric Force in the Half-Squat Exercise

MIF was assessed in the HS exercise executed on a Smith machine (Hammer Strength Equipment, Rosemont, IL, USA). The body position in the test was validated by an experienced test administrator who set the bar on the safety pins at a height corresponding to 90° of knee flexion, as established during the pre-training testing sessions. The same bar height was repeated at the post-training test for each athlete. Throughout the measurements, after a starting command, participants applied force as rapidly as possible against the mechanically fixed bar for 5 s. The peak force was determined using a force plate with custom-designed software (Kistler Quattro Jump; Kistler Instrument Corp, Winterthur, Switzerland), which sampled at a rate of 1000 Hz. The force plate was fixed to the floor using a specific and standardized base. Strong verbal encouragement was provided across all attempts, and data were normalized by dividing the absolute force values by the athletes’ BM (i.e., relative force = N^.^kg^−1^).

#### Progressive Loading Test in the Jump-Squat and Half-Squat Exercises

PP was measured in the JS and HS exercises performed on a Smith machine (Hammer Strength Equipment, Rosemont, IL, USA), as previously described (Loturco et al., 2017, 2022a). Players were required to execute three repetitions at maximal velocity for each load, with a 5-min rest interval provided between sets. The test started at a load corresponding to 40% of the athlete’s BM and a load of 10% BM for all exercises was gradually added up to 100% BM (Loturco et al., 2022a). To determine power output, a linear velocity transducer (T-Force, Dynamic Measurement System; Ergotech Consulting S.L., Murcia, Spain) sampling at 1000 Hz was attached to the barbell. The maximum PP values obtained for each load and exercise were used for analysis. Data were normalized by dividing the absolute power values by the athletes’ BM (i.e., relative power = W·kg^−1^). The HS-1RM, which served as the variable for the JS loads in each group, was estimated based on the peak velocity values obtained at the 100% BM load using the formula previously described by Martínez-Cava et al. (2019).

### Statistical Analysis

Data are presented as mean ± standard deviation. Data normality was checked using the Shapiro-Wilk test. Two-way ANOVA with repeated measures (group*time) followed by the Tukey’s post-hoc test was used to examine pre- and post-differences between groups. The level of significance was set at *p* < 0.05. Absolute and relative reliability were assessed using the coefficient of variation (CV) and the two-way random intraclass correlation coefficient (ICC), respectively. To determine the magnitude of the differences between pre- and post-training data and delta changes, effect sizes (ES) along with their 95% confidence intervals (CI) were calculated and interpreted using the thresholds proposed by Rhea (2004) for highly-trained subjects, as follows: <0.25, 0.25–0.50, 0.50–1.00, and >1.00 for trivial, small, moderate, and large, respectively.

## Results

**Figure 1:**
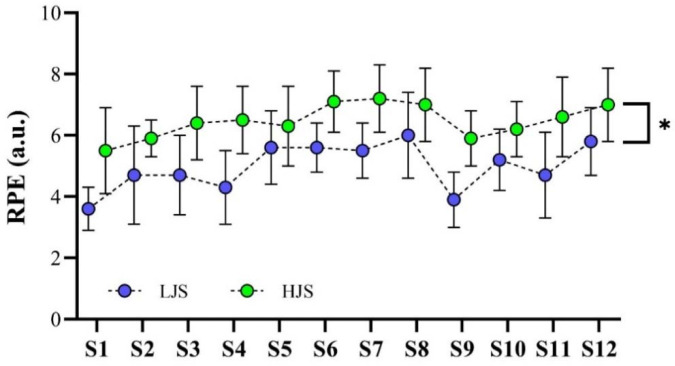
Variations in the session rating of perceived exertion (RPE) across the 12 resistance training sessions (S) for both training groups. *p < 0.05 for all training sessions.

All measurements used in this study exhibited high levels of absolute and relative reliability (i.e., ICC > 0.90 and CV < 10%). No significant differences between groups were observed for any variables assessed in the baseline testing battery (*p* > 0.05). Figure 1 shows the variations in the RPE scores across the 12 resistance training sessions in both light- and heavy-load JS training groups. The HJS group reported higher RPE values than the LJS group in all resistance training sessions (*p* = 0.013 for the main effect of group).

**Figure 2:**
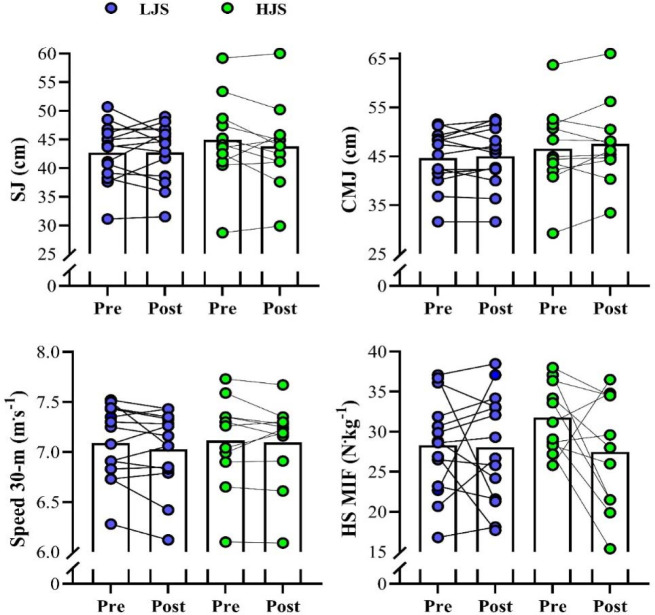
Variations in the squat and countermovement jumps (SJ and CMJ), 30-m sprinting speed, and maximum isometric force (MIF) in the half-squat (HS) exercise for the light-load jump-squat (LJS) and heavy-load jump-squat (HJS) groups during the 4-week training period.

Figure 2 depicts the comparisons of the vertical jump, sprint speed, and MIF at the pre- and post-training for both groups. No significant changes were detected for the SJ (ES [95%CI] = 0.01 [−0.74; 0.74]; 0.13 [−0.71; 0.96] for LJS and HJS groups, respectively; *p* > 0.05), the CMJ (ES [95%CI] = 0.07 [−0.67; 0.81]; 0.12 [−0.72; 0.95] for LJS and HJS groups, respectively; *p* > 0.05), and 30-m sprint speed (ES [95%CI] = 0.16 [−0.58; 0.90]; 0.07 [−0.77; 0.90] for LJS and HJS groups, respectively; *p* > 0.05). For MIF, although no significant changes were detected between pre- and post-training in both groups (*p* > 0.05), the HJS group experienced a large and meaningful (i.e., the lower bound of the CI did not cross “0”) decrement (ES [95%CI] = 1.01 [0.08; 1.84]; which represented a 14% decrement in the MIF value), while a trivial change was observed for the LJS group (ES [95% CI] = 0.05 [−0.69; 0.79]; representing only a negligible variation of −1%).

**Figure 3:**
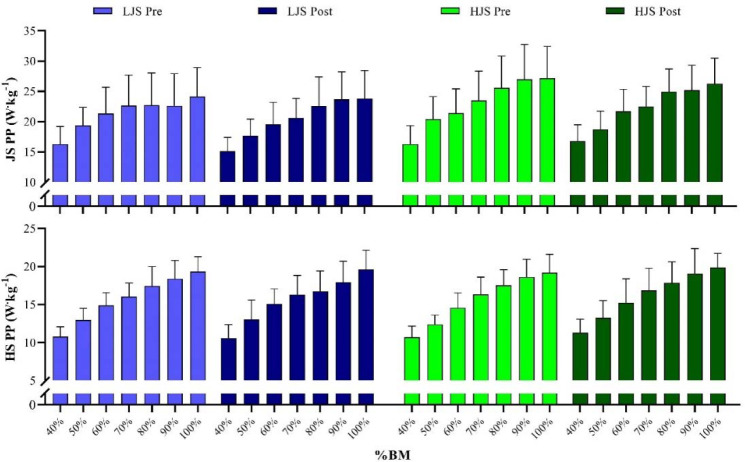
Variations in the half-squat (HS) and jump-squat (JS) peak power (PP) output over the different loads tested for the light-load jump-squat (LJS) and heavy load jump-squat (HJS) groups during the 4-week training period. BM: *body mass*.

Figure 3 shows the changes from pre- to post-training in PP for both JS and HS exercises, across the range of loads, within both LJS and HJS training groups. No significant differences were observed for JS-PP (ES [95%CI] ranging from 0.02 [−0.72; 0.76] to 0.57 [−0.20; 1.30], and from 0.08 [−0.76; 0.91] to 0.43 [−0.43; 1.26] for LJS and HJS groups, respectively; *p* > 0.05), as well as for HS-PP (ES [95%CI] ranging from 0.01 [−0.74; 0.74] to 0.23 [−0.52; 0.97], and from 0.19 [−0.65; 1.02] to 0.75 [−0.14; 1.58] for LJS and HJS groups, respectively; *p* > 0.05).

## Discussion

We investigated the effects of two different JS training schemes executed under different loading conditions (i.e., light vs. heavy loads) integrated into the real-world training routine of elite rugby players. The main finding of this study was that heavy loads during JSs (80% HS-1RM) did not provide any additional benefits on strength, power, and speed performance than those induced by lighter JS loads (40% HS-1RM). This pattern was observed with the significant advantage that lighter JS loads resulted in lower levels of perceived exertion (i.e., the RPE) in all resistance training sessions. Notably, these sessions were completed over a 4-week training phase, coinciding with the initial phase of the competitive training period.

A previous study (Valenzuela-Barrero et al., 2023) utilized the same loading ranges to compare the effects of light (40% 1RM) and heavy (80% 1RM) loads in the full squat exercise in recreationally trained men (n = 22) and women (n = 16) over a period of six weeks. Despite some important differences between the two experimental designs (i.e., a JS vs. a full squat, a sample composed of men and women, and the same relative volume load [sets x reps x % 1RM]), in general, both loading conditions yielded similar strength gains for men and women. However, certain trends suggest minor differences between groups. In that study, according to standardized mean differences, heavier loads seemed to lead to greater adaptations in maximum strength in men, while women appeared to benefit equally from both light and heavy loading conditions (Valenzuela-Barrero et al., 2023). Whereas lighter loads (40% 1RM) may be more suitable for enhancing force application in activities involving only body weight as a workload (such as jump tasks), this potential advantage might be compromised in individuals with greater strength (i.e., males) who tend to exhibit more aggressive deceleration towards the end of the lift (Loturco et al., 2022b, 2023; Valenzuela-Barrero et al., 2023). In theory, this drawback could be avoided (or at least reduced) with the use of ballistic exercises (e.g., a JS) which allow for continued acceleration throughout the entire range of motion up to the projection point (e.g., jump trial) (Cormie et al., 2011a). This continued acceleration will also result in higher force, power, and velocity output along with lower levels of strength deficit (when compared to traditional HSs executed at the same loads) (Loturco et al., 2023).

The strength deficit is essentially a measure of the difference between the force generated at the 1RM and any other submaximal force value (Gonzalez-Badillo et al., 2017; Loturco et al., 2021; Zabaloy et al., 2022). Put differently, this force-derived measurement may indicate the ability that a given subject has to apply substantial amounts of force against lighter loads, at higher velocities (Loturco et al., 2021). It was observed, for example, that elite sprinters exhibited lower levels of strength deficit and demonstrated superior sprint, jump, and relative strength performance than professional rugby players (Loturco et al., 2021), thereby confirming their capacity to generate larger forces against their own body mass (Loturco et al., 2021; Zabaloy et al., 2022). Another study exclusively conducted with professional rugby players (Cunningham et al., 2013) also highlighted the importance of relative strength and power metrics in the appropriate development of various speed components, such as acceleration and maximal speed (i.e., top-speed). Specifically, an investigation with elite young rugby players (under-20) (Zabaloy et al., 2022) revealed that strength deficit and relative strength in the back squat exercise were closely related to a series of speed-power measures, irrespective of playing positions (i.e., backs and forwards). Considering these mechanical aspects, the results of previous studies, and the potential adaptations provided by ballistic exercises performed with relatively lighter loads (e.g., positive adaptations in the neural drive, the rate of neural activation, and inter-muscular coordination specific to sport-specific activities) (Cormie et al., 2011a, 2011b; Loturco et al., 2023; Mcbride et al., 2002), it was expected that the LJS group would achieve greater gains in unloaded and faster movements than the HJS, such as sprint and jump tasks. Nevertheless, both light and heavy JS loads were incapable of improving vertical jump and sprint speed abilities in elite rugby players during the competitive period. It is worth noting that the JS programs were completed along with other complementary resistance exercises (Table 2), which were part of the regular resistance training routine for rugby players. Hence, these additional exercises, executed under mixed loading conditions (i.e., 60–90% 1RM), could have hampered or at least constrained the potential different adaptations produced by both JS loads.

These effects become clearer when comparing, for example, the current results with those of Mcbride et al. (2002). In that study, the authors compared, in an isolated manner, (i.e., performing only the JS exercise throughout the intervention period) the effects of heavy- vs. light-load JS on the development of strength, speed, and power. For those authors, according to their findings, “the velocity of the movement, as controlled by the load, plays a key role in improving high-velocity performance capabilities and possible neural mechanisms of adaptation” (Mcbride et al., 2002). Therefore, the light-load group obtained better results in speed-related tests (i.e., a trend towards improved 20-m sprint speed), whereas the heavy-load group showed a significant decrease in 5-m sprint speed. In contrast, interestingly, the subjects who trained under either lighter or heavier loading conditions (i.e., 30% and 80% HS-1RM, respectively) demonstrated similar maximum strength gains in the squat exercise (Mcbride et al., 2002). Remarkably, in both studies, the subjects (e.g., recreationally trained individuals or professional athletes) were instructed to accelerate upwards as fast and forcefully as possible, aiming to achieve maximum height and, thus, apply the maximum amount of force while jumping. This confirms the importance of movement velocity in promoting positive adaptations, not only for the development of sport-specific performance, but also for inducing specific gains in strength-related qualities (González-Badillo et al., 2014; Mora-Custodio et al., 2016; Pareja-Blanco et al., 2014).

In fact, the rationale for the use of heavy-loading conditions for the optimization of maximum strength (and other physical capabilities) is not new and is based on a simple and traditional concept: the activation of fiber types during a muscular contraction is influenced by the level of force exerted (Young, 1993). Slow motor units are recruited for low-force contractions, and as the required force increases, fast motor units are simultaneously recruited (Kraemer and Newton, 2000; Newton and Kraemer, 1994; Young, 1993). Hence, it is reasonable to suppose that the activation of the fastest high-threshold motor units may necessitate the utilization of heavier loads (moved at slower velocities), as only these loads ensure a maximum voluntary contraction (Kraemer and Newton, 2000; Newton and Kraemer, 1994; Young, 1993). Nonetheless, in this study, for elite rugby players training and competing over the first phase of the competitive season, these precepts did not prove to be a significant factor; conversely, the HJS group reported higher levels of perceived fatigue, as demonstrated by their superior values of the RPE (Figure 1). This may be a critical issue in elite athletes since, as a general pattern, they train two or more times per day, which results in limited time for recovery between successive training sessions (and competitions) (Tavares et al., 2017). Moreover, in addition to the reasons mentioned above (i.e., increased risk of non-functional overreaching, muscle pain, injuries associated with reduced muscular coordination, and decreases in athletic performance) (Moreno-Villanueva et al., 2022; Ribeiro et al., 2022; Schmitz et al., 2014), several studies have found close correlations between internal measures of training loads (i.e., the RPE) and “subjective wellness” with independent measures of athletic performance (Day et al., 2004; Hills and Rogerson, 2018; Nakamura et al., 2016; Pereira et al., 2022b). For example, Hills and Rogerson (2018) observed a range of strong relationships between numerous CMJ variables (i.e., peak velocity, time to peak velocity, and CMJ duration) and self-reported well-being, as assessed by custom-designed short-form questionnaires, in professional rugby players. Overall, positive correlations were detected between higher levels of perceived well-being and CMJ velocity and time to peak velocity (r ≈ 0.70, for both correlations), whereas negative correlations were obtained between these subjective measures and CMJ duration (r = −0.67). In simpler terms, neuromuscular fatigue could modify stretch-reflex sensitivity and decrease muscle-tendon stiffness, which, among other neuromechanical adjustments, might lead to an increase in CMJ duration (and a decrease in CMJ performance). From a practical perspective, this suggests that wellness scores and perceived exertion, even when evaluated through practical and applied subjective reports, appear to be sensitive in detecting neuromuscular fatigue and, consequently, changes in neuromuscular performance. Unquestionably, this outcome (i.e., lighter loads producing similar results to heavier loads in rugby players associated with lower levels of the RPE) has important implications in real training settings due two main reasons: (1) the RPE method has already proven to be a reliable and useful tool for researchers and coaches to assess exercise intensity during resistance training (Day et al., 2004); (2) the vast majority of rugby coaches working within professional rugby union environments consider the session-RPE a valid and effective tool for monitoring and managing training loads (Comyns and Hannon, 2018).

Perhaps the most significant finding of this study was that, despite training with twice the load (i.e., 40% vs. 80% HS-1RM), the HJS group did not achieve better results in maximum strength, speed, and power tests. Worse still, they experienced a significantly higher level of perceived effort (as assessed by the RPE) throughout the 4-week intervention, conducted within the first phase of the competitive period. This occurrence may pose a significant challenge for team-sport disciplines since, when combined with other typical issues that commonly arise across the season (e.g., injuries, muscle pain and soreness, and illnesses), accumulated fatigue can have a detrimental impact on physical, technical, and tactical performance (Moreno-Villanueva et al., 2022; Ribeiro et al., 2022; Thorpe et al., 2017).

This study is limited by several factors, most of which are related to the difficulties and constraints imposed by elite athletes’ training settings. First, the JS training programs were implemented in conjunction with the complementary resistance training routines, rugby-specific training sessions, and matches. Therefore, similarly to any study executed in real-world contexts, it is not possible to state that the results obtained herein are exclusively associated with the heavy or light JS training schemes. Lastly, the short-duration (i.e., 4 weeks) of the intervention precludes us from drawing firm conclusions concerning the effects of lighter or heavier loading conditions (specifically employed in ballistic exercises) on the physical and technical qualities of elite rugby players. On the other hand, the fact that considerably lighter loads (representing 50% of the absolute load; i.e., 40% vs. 80% HS-1RM) have the same effects as much heavier loads when used by very strong athletes (1RM relative to BM ≥ ~2) in loaded jumps, and still result in lower levels of fatigue, especially within the competitive period, can be viewed as a relevant contribution of this study. Future studies incorporating longer interventions (i.e., 6–10 weeks) at different phases of the competitive season and using various methods and tools to detect performance changes (e.g., significant changes in strength, speed, and power performances) along with fatigue-related symptoms and variables are necessary to either refute or consolidate the findings presented here.

## Conclusions

When performed during the initial phase of the competitive period, both light- (40% HS-1RM) and heavy-load (80% HS-1RM) JSs have similar effects on the physical performance of elite rugby players. Despite these apparent similarities, two crucial aspects should be considered by coaches and practitioners when prescribing JS overloads throughout this important training phase: (1) although non-significant, the differences in favor of light loading conditions (as demonstrated by the 14% decrement in the MIF and an ES equal to 1.01 in the HJS group; whereas the LJS group practically maintained their strength level) can be seen as a key aspect when selecting the best loading ranges in the course of the competitive period; (2) the lower level of perceived effort (i.e., RPE) exhibited by the LJS group for every resistance training session may also be regarded as a decisive factor to determine the more adequate loading intensities for these elite athletes, who usually report and present higher levels of fatigue in response to rugby-specific training sessions and matches. Rugby coaches and their technical staff should carefully observe these trends when designing log-term strength-power training programs for elite rugby players, especially when defining the most effective loading intensities for prescribing ballistic exercises.
